# Equitable evaluation of supply-demand and layout optimization of urban park green space in high-density linear large city

**DOI:** 10.1371/journal.pone.0310015

**Published:** 2024-09-06

**Authors:** Shibao Yu, Peng Zeng, Xiaoling Xie, Dandan Chen

**Affiliations:** 1 School of Architecture and Urban Planning, Lanzhou Jiaotong University, Lanzhou, Gansu, China; 2 School of Architecture, Tianjin University, Tianjin, China; 3 Lanzhou Urban & Rural Planning and Design Institute, Lanzhou, Gansu, China; Zhejiang A&F University, CHINA

## Abstract

Equitable and effective planning of urban park green spaces (UPGSs) is an important way to promote green and healthy urban development and improve citizens’ quality of life. However, under the background of rapid urbanization, linear large cities, with their unique spatial forms and high-density population agglomerations, have brought special challenges for the planning and management of urban public green spaces. This study takes Lanzhou, a typical representative of high-density linear large cities in China, as a case study. Based on the improvement of the traditional Gaussian Two-Step Floating Catchment Area method (G2SFCA), combined with the Gini coefficient and the Lorentz curve, the social equity and spatial equity of UPGS supply-demand in the central urban area of Lanzhou were evaluated at the city and district scales. Meanwhile, the areas with shortage of UPGS supply-demand were accurately identified as the key areas for future optimization. The results show that: (1) There are significant differences in the equity of UPGS supply-demand in the linear large Lanzhou at the social and spatial levels, and most UPGS resources are enjoyed by a few people; (2) The spatial accessibility of UPGSs has an obvious “string of beads” distribution Characteristics, and the areas with high accessibility are mainly concentrated along rivers; (3) The equity of UPGS supply-demand exhibits a spatial gradient effect, which is characterized by a circle distribution. From the inside to the outside, it is as follows: good supply—dense population, good supply—sparse population, supply shortage—dense population, supply shortage—sparse population. Finally, based on the concept of “progressive micro-regeneration” and the Location Allocation model (LA), the optimal sites for new UPGSs were determined, maximizing the equity of UPGS supply-demand. This provides a practical reference for relevant management departments to optimize park layouts in the future.

## Introduction

Under the challenges of climate change, public health events and rapid urbanization, the urban ecological environment is receiving increasing attention. As an important part of the urban ecosystem, urban park green spaces (UPGSs) can not only improve the quality of urban environment by purifying air, attenuating noise and decreasing temperature, but also provide social and entertainment venues, promote public health and increase human well-being [1, 2]. The World Health Organization (WHO) has also proved that UPGSs have beneficial effects on people’s health and well-being through a large amount of evidence [3]. As UPGSs can provide diverse ecosystem services, improve the quality of life, and benefit physical and mental health, urban residents and managers are increasingly aware of its importance.

The linear city is an urban planning concept first proposed by Spanish engineer Soria y Mata in 1882. The idea of linear city is to emphasize that the city mainly develops along slender traffic lines. Compared with the traditional radial or grid-like urban development model, the spatial structure of linear cities shows unbalanced characteristics. When the city is large and the population and building density are high, this extremely unbalanced spatial structure often causes traffic congestion in the main development direction of the city and inconvenient connection between various functional land uses in the city [4, 5], which also brings great challenges to the planning and management of urban green space system. The United Nations usually classifies cities with 100,000 to 1,000,000 people as large cities, while China classifies cities with 1–5 million permanent residents in Urban area as large cities. For high-density cities, there is no uniform standard in the world. Relevant research believes that the threshold standard for high-density cities in the world is 15000 people/km^2^ [6]. Therefore, in this paper, the high-density linear large cities are defined as cities with urban population and population density of about 1–5 million and 15000 people/km^2^ respectively, and mainly developed along long and narrow transportation lines. Due to the more serious contradiction between supply and demand of UPGSs in high-density linear large cities, it is urgent to reveal the supply and demand characteristics of UPGSs in high-density linear large cities through relevant research, so as to formulate effective planning strategies.

The concept of environmental justice emerged in the 1970s, which not only emphasizes the equitable use and distribution of environmental resources, but also emphasizes the equity of the impact of environmental quality on different social groups [7]. A comprehensive concept of global environmental justice should be locally grounded, theoretically broad, and include diverse issues such as distribution, procedures, recognition, participation, and capabilities [8, 9], especially the spatial distribution of environmental resources as a typical environment justice issue has always been the focus of people’s attention [10–12]. Over the past few decades, scholars from various countries have conducted a lot of research on the equitable allocation of UPGSs in space. Some scholars have evaluated the spatial distribution of UPGSs to determine whether the distribution of UPGSs is equitable, and these assessments mainly include indicators such as green space coverage, green space density, and green space accessibility [13, 14]. Some scholars have considered the impact of the economic status, social class, race and age of different social groups on the demand for green space resources [15–18], to ensure that green space resources should be able to meet the needs of different groups. Some scholars have also considered the impact of the quality and service level of green space o on the equitable utilization of UPGSs [19, 20], to ensure that the quality and service level needs of all community green spaces are met and to avoid the gap between the rich and the poor and social exclusion. However, we will mainly focus on the allocation equity of UPGSs in high-density linear large cities from the perspective of urban morphology.

Scientific evaluation of supply-demand equity is the basis for rational allocation of UPGSs. Barrier-free spatial accessibility has been considered as one of the important indicators to quantify equity[21, 22]. Currently, the methods to evaluate the accessibility of UPGSs mainly include survey research method, coverage analysis method, travel cost analysis method, space syntax, mathematical model and gravity model. The survey research method mainly relies on field surveys[23, 24], interviews and questionnaires [25, 26], and user participation [27]. This method has limitations due to its subjectivity. The coverage analysis methods evaluate the accessibility of parks by measuring the number, area, or density of parks in a specific area [28, 29], or based on their spatial location [23, 30, 31] However, this method usually simplifies the calculation of distance and fails to adequately consider actual road conditions. The travel cost analysis method uses GIS network analysis tools [32, 33] or open map API interfaces [34, 35] to obtain the travel cost of different transportation modes and evaluate the accessibility of UPGSs. This method is more accurate and objective, but it requires high-quality data and is limited by the number of API interfaces. Spatial syntax evaluates the accessibility of urban parks from the perspective of spatial topological relationships [36–38], which highlights the impact of spatial structure or form on park accessibility. However, it overlooks factors such as park type and scale, and the distance from residential areas. The mathematical model constructs more accurate models by combining factors such as traffic distance, land use, building environment, and socio-economic characteristics. For example, the green space accessibility (GSA) index is combined with Lorenz Curve, Gini Coefficient [39], the expansion method [40] and the comprehensive index method [41]. This method is more detailed and targeted, such as walking accessibility of children and the elderly, perceived accessibility, but it requires higher accuracy and quality of data. The gravity model is currently a popular park accessibility evaluation method. It is based on the theory of spatial interaction, considering factors such as supply, demand, and spatial distance to measure accessibility [42]. The Two-Step Floating Catchment Area (2SFCA) method proposed by Radke and Mu (2000) is a special method and a typical representative of the gravity model. Considering that the traditional 2SFCA model ignores the distance attenuation effect within the search range, Dai introduced the Gaussian function in 2011 to improve the traditional 2SFCA model, also known as the Gaussian Two-Step Floating Catchment Area method (G2SFCA), which makes up for the above defects [43]. Later, based on the traditional G2SFCA model, there were many improved models that consider factors such as search radius [44], travel mode [45], supply-demand adjustment [46], travel behavior [47], population [48, 49], actual travel cost and travel experience [50]. These improvements further enhanced the scientific accuracy of accessibility measurement. The first five methods mentioned above primarily evaluate park accessibility through surveys and questionnaires, statistical indicators of the parks themselves, travel costs, spatial topological relationships, and mathematical models. Either there is a strong subjectivity in data acquisition or the setting of some model parameters, or the accessibility factors considered are relatively simple, resulting in a lack of rationality in the evaluation results. The G2SFCA method is widely used because it fully considers the supply-demand relationship, and can also add relevant parameters or optimize the original parameters according to the actual situation of the research problem. Therefore, this study also adopts the G2SFCA method to evaluate the accessibility of UPGSs.

Although many studies have improved the traditional G2SFCA method, more factors such as travel distance, search radius, and travel mode have been considered, while less consideration is given to factors such as residents’ subjective choice willingness and the surrounding environment of the park. Meanwhile, the threshold value is a key factor in the measurement of UPGS accessibility, and different thresholds should be applied for various transportation modes and parks of different scales [45, 51]. Therefore, the subjective choice of residents, the diversity of the park’s surrounding environment, and multi-level time thresholds should be considered in the measurement of UPGS accessibility. Moreover, existing research often uses relative values for supply-demand ratios [52–54], which can lead to overly large or small results for supply-demand equity in different cities. Countries like China will carry out macro-control of urban resource allocation, which will undoubtedly further increase the equity gap of resource allocation. Taleai, M et al. proposed a supply-demand equity model based on minimum service standards, which considers not only the different service standards of different facilities, but also the uniform standards of the same facility in different regions [55]. Therefore, this study also adopts the modeling approach of minimum service standards to measure the equity of UPGS supply-demand. In addition, under the conditions of limited urban land resources, there is an urgent need for a scientific method to add UPGSs and improve the accessibility and quality of UPGSs to ensure that everyone in the city has equal opportunities to enjoy good environmental resources. The Location Allocation (LA) model based on GIS technology has been proven to effectively solve the problem of reasonable distribution and layout optimization of UPGSs under limited conditions [56–58].

Under the above background, this study mainly addresses the following three specific issues: (1) Improving the traditional G2SFCA model through parameter optimization to scientifically evaluate the accessibility of UPGSs;(2) Revealing the accessibility and supply-demand equity characteristics of UPGSs in high-density linear large cities; (3) Scientifically determining the optimal locations for new UPGSs under the condition of limited urban land resources, to maximize the equity and efficiency of UPGSs in high-density linear large cities. Based on the above problems and the limitations of the traditional G2SFCA method in the review, we took Lanzhou, a typical representative of China’s high-density linear large cities, to conduct a detailed study, which provides a reference for similar cities and relevant departments to fairly allocate UPGS resources.

## Materials and methods

### Study area

Lanzhou City (102°36′–104°35′ E and 35°34′–37°00′ N), located in the northwest region of China, is the capital of Gansu Province and one of the important central cities in Western China. The population density and urbanization level are at the forefront of the cities in the northwest region. By the end of 2021, Lanzhou had a total area of 13,100 km^2^, with a population density of 335 people per square kilometer, and an urbanization rate of 84%. Meanwhile, the north and south sides of Lanzhou City are surrounded by mountains, and the Yellow River crosses from the center of the city, making it a typical linear city with a distinct landscape of mountains and water. However, located deep in the northwest inland area with less precipitation, a dry climate, and a fragile ecology, Lanzhou has limited green spaces. The city’s urban land is extremely scarce due to the constraints of the mountains to the north and south, leading to a trend of encroaching on green spaces. By the end of 2020, the green coverage rate in the built-up area was 40.03% (the national standard for Chinese garden cities is ≥41%), and the per capita UPGS in municipal districts was 9.1 m^2^ (the standard for Chinese garden cities ≥ 12 m^2^). This indicates that the level of greening in Lanzhou’s municipal area is still relatively low, and the supply-demand conflict of UPGSs in the central urban area is even more acute. Therefore, it is urgently necessary to conduct research on the spatial allocation equity of UPGSs and its influencing factors, providing references for government departments to equitably allocate UPGSs.

**Fig 1:**
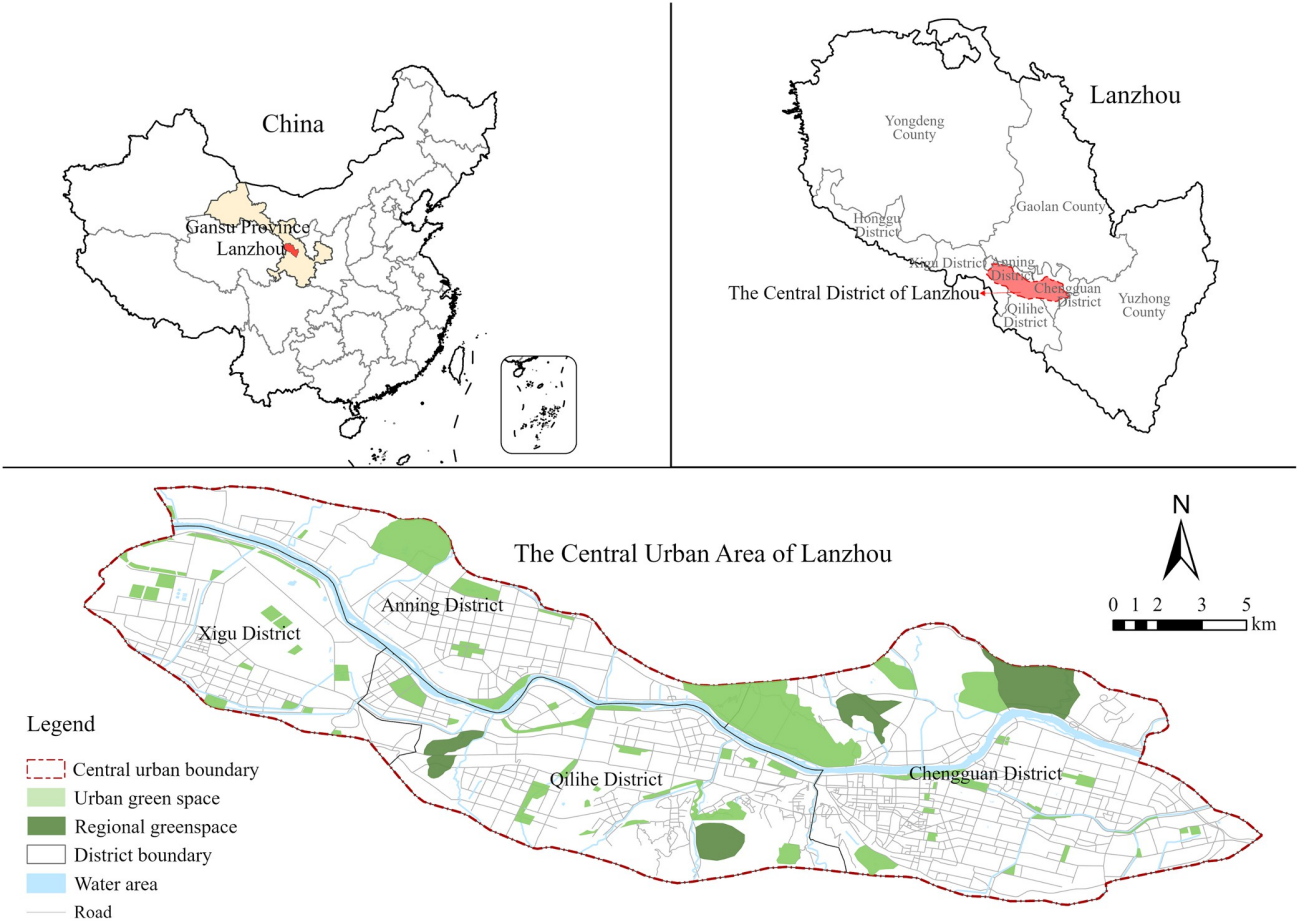
Overview of the study area.

The main scope of this study is the central urban area of Lanzhou as designated in the “Lanzhou City Master Plan (2011–2020)”. The study area mainly covers Xigu, Anning, Qilihe and Chengguan districts, involving 48 streets with a total area of about 217.33 km^2^. In order to visually display the research scope of Lanzhou’s central urban area, we drew the following location map based on OpenStreetMap and combined with field surveys (Fig 1).

### Technology flowchart

**Fig 2:**
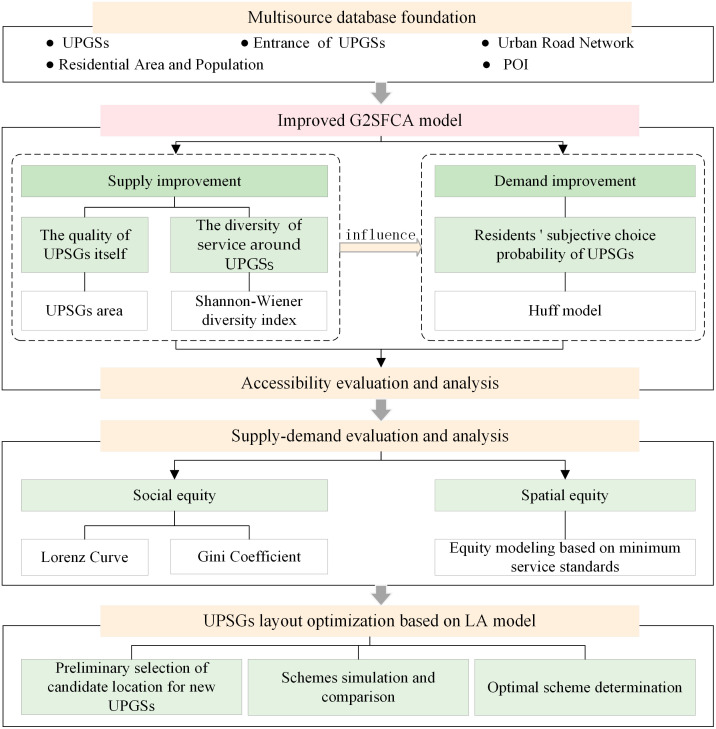
Technology flowchart.

This study has established a research framework for UPGS accessibility assessment and layout optimization by combining the improved G2SFCA method and the LA model (Fig 2). Firstly, the UPGSs in the central urban area of Lanzhou City were selected as the research object, and the research database was established with the help of multi-source big data such as land use data, road traffic data, park entrance points, POI and residential population. Secondly, addressing the limitations of the traditional G2SFCA method in neglecting residents’ subjective willingness to choose visiting parks and the diversity of service around UPGSs, improvements were made from both the supply and demand aspects. Simultaneously, a multi-level time threshold based on the concept of living circles was set. Furthermore, the improved G2SFCA method was used to evaluate the accessibility of UPGSs, and the equity of UPGS supply-demand was evaluated from the two dimensions of social equity and spatial equity. The supply shortage areas were accurately identified. Finally, combined with the existing urban stock land use status and the layout requirements of different UPGSs, the potential locations of the newly added urban parks were selected, and the optimal layout plan was determined by the LA model, which provides a reference for optimizing the layout of UPGSs.

### Data sources and processing

This paper collected and organized the data of UPGSs, road traffic, water area, population and POI in Lanzhou City, and established a multi-source basic database based on the GIS platform. We declare that the above data used in this paper were collected through the open platform OpenStreetMap and by the authors through field research and hand mapping, and do not involve copyright issues. To improve the reproducibility of this work, we provide a URL to obtain a dataset that reproduces this work in the data availability section. The sources and collection methods of each dataset are described in detail below:

#### Data of UPGSs

**Fig 3:**
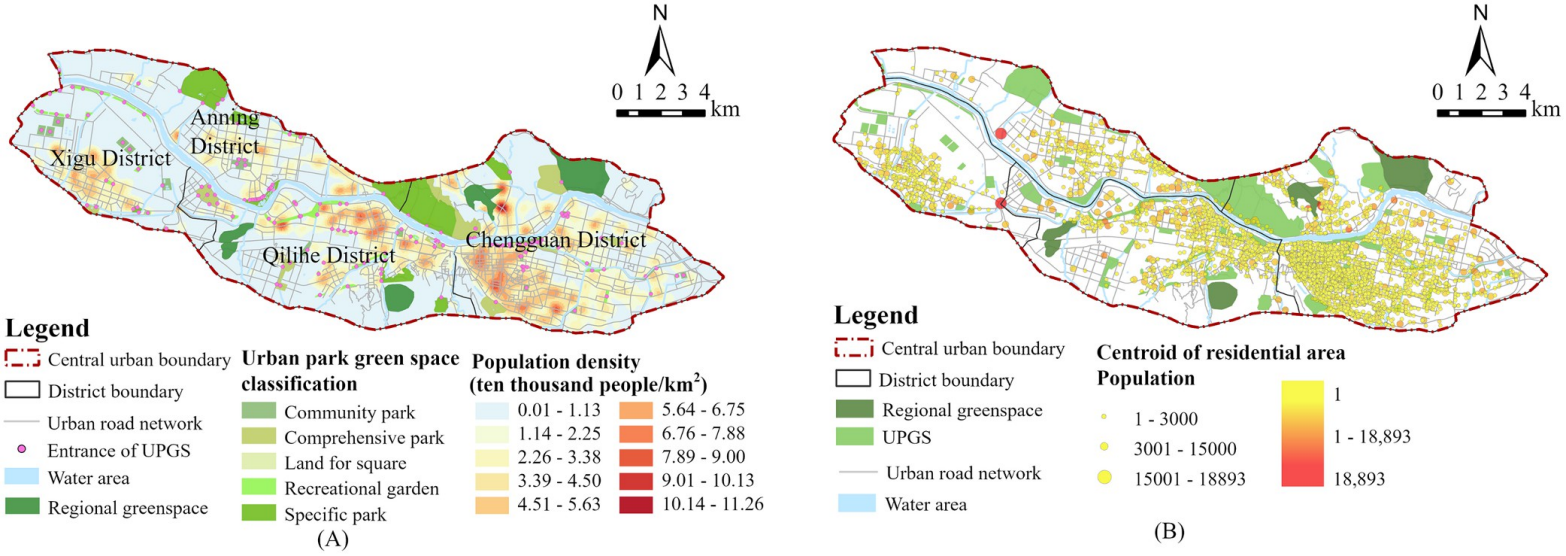
(A) Spatial distribution of the UPGSs, population density and road network; (B) Spatial distribution of residential points and population.

According to the “Urban Green Space Classification Standard (CJJ/T 85–2017)”, four types of UPGSs (G1) were selected as the research objects. These types include comprehensive parks (G11), community parks (G12), specific parks (G13), recreational gardens (G14), as well as square land with a green rate of more than 65% (G3). Comprehensive parks (G11) refer to the green space with rich content, suitable for various outdoor activities, perfect recreational and supporting management facilities, and the area is generally larger than 10ha. Community parks (G12) refer to the green space with independent land use, basic recreational and service facilities, mainly for residents within a certain community to carry out daily leisure activities nearby, and the area is generally greater than 1ha. Specific parks (G13) refer to the green space with specific content or form and corresponding recreational and service facilities, such as zoos, botanical gardens, heritage parks, etc. Recreational gardens (G14) refer to the green space with independent land use, small scale or diverse shapes, convenient for residents to enter the nearest people, and certain recreational functions in addition to the above various parks. According to OpenStreetMap and field surveys, we finally manually drew the vector data of 89 UPGSs, with a total area of about 21.19 km^2^, excluding vector polygons with an area less than 0.2 hm^2^ and a width less than 12 m (Fig 3A, Table 5).

#### Entrance data of UPGSs

This study takes the real entrance of the UPGSs as the supply point. However, considering that recreational gardens and some specific parks often do not have clearly defined entrances or have multiple entrances for the convenience of citizens, and their scale is relatively small, combined with the principle of data availability, the entrance to these parks still takes its geometric center of gravity as the supply point. According to OpenStreetMap and field surveys, we finally identified 169 UPGS entrance points as supply points (Fig 3A).

#### Data of urban road network

According to OpenStreetMap, we obtained the road network and water system data of Lanzhou’s central urban area and established the urban road network dataset in ArcGIS. The road network dataset shows topological relationships, with intersections and roads abstracted as nodes and links respectively, and the time spent moving on the road as resistance, thus realizing the simulation of the real paths for residents to reach UPGSs (Fig 3A). The road networks and water systems in the cities of Taiyuan and Xi’an were also mapped according to OpenStreetMap (Fig 9).

#### Residential area and population data

According to OpenStreetMap, we obtained the location points of 3853 residential areas in Lanzhou’s central urban area and the height, number of floors, and type of residential buildings in each residential area, and estimated the total number of households in each residential building. In addition, according to the “2020 China City Statistical Yearbook”, it can be calculated that the average population per household in Lanzhou City is 2.7, and the population of each residential area can be estimated by multiplying the total number of households by the average number of people per household. Finally, the estimated residential population was calibrated using the township (street) population data in the seventh national census, and the calibrated residential population was used to indicate the scale of its demand for UPGSs (Fig 3B). The population density was calculated by the kernel density analysis tool in ArcGIS (Fig 3A). Compared to previous studies that used district centroids and population data, we provide the necessary support for UPGS accessibility analysis at the fine scale.

#### POI data

POI data for the central urban area of Lanzhou was obtained through the OpenStreetMap platform, which is divided into 17 categories: catering services, scenic spots, public facilities, companies and enterprises, shopping services, transportation facility services, financial and insurance services, science and education cultural services, motorcycle services, automobile services, automobile maintenance, automobile sales, life services, sports and leisure services, government institutions and social organizations, medical and healthcare services, and accommodation services. We constructed the road traffic network model in ArcGIS based on Lanzhou’s road network dataset. Combined with the service radius and time cost thresholds for different levels of UPGSs as listed in Table 1, the service areas for UPGSs were established, and the POI points within the service range of each UPGS were filtered out. Subsequently, the Shannon-Wiener diversity index was used to calculate the diversity index of urban POI within the service range of UPGSs.

**Table 1:** Resource allocation of different park types in the Central Urban Area of Lanzhou.

Study Area	Community Park		Comprehensive Park		Land for Square		Recreational Garden		Specific Park		Total	
	Number / unit	Total Area / hm^2^	Number / unit	Total area / hm^2^	Number / unit	Total area / hm^2^	Number / unit	Total area / hm^2^	Number / unit	Total area / hm^2^	Number / unit	Total area / hm^2^
Anning District	4	28.76	--	--	--	--	4	17.06	4	864.65	12	910.47
Chengguan District	6	29.50	5	401.71	5	16.66	7	29.12	5	117.78	28	594.77
Qilihe District	2	22.37	5	124.55	1	16.85	15	121.52	2	97.49	25	382.78
Xigu District	6	104.70	1	19.60	--	--	16	98.03	1	8.23	24	230.57
Total	18	185.33	11	545.86	6	33.52	42	265.72	12	1088.15	89	2118.58

## Methods

### UPGS supply-demand evaluation method

#### Traditional G2SFCA method

The Two-Step Floating Catchment Area method (2SFCA) was initially proposed by Radke and Mu (2000) [59], which is an accessibility calculation method based on spatial interaction. Its principle involves two search calculations for supply points and demand points respectively. Due to its strong operability and ability to accurately reflect supply-demand relationships, it is widely used in calculating the accessibility of various public service facilities, including urban parks. However, the 2SFCA method has its limitations, such as neglecting the distance decay effect within the search range. The Gaussian function has significant advantages in simulating distance decay effects. To address this shortcoming, Dai introduced the Gaussian function into the traditional 2SFCA model in 2011, also known as the Gaussian Two-Step Floating Catchment Area method (G2SFCA) [43]. The calculation steps are as follows:

$${\text{R}}_{\text{j}}=\frac{S_{j}}{\sum_{k\in \left\{d_{kj}\leq d_{0}\right\}}\;G\left(d_{ij}\right)D_{k}}$$ (1)

$$G\left(d_{ij},d_{0}\right)=\left\{\begin{array}{cc} \frac{e^{-\left(\frac{1}{2}\right)\times {\left(\frac{d_{ij}}{d_{0}}\right)}^{2}}-e^{-\left(\frac{1}{2}\right)}}{1-e^{-\left(\frac{1}{2}\right)}}, & d_{kj}\leq d_{0}\\ 0, & d_{kj}>d_{0} \end{array}\right.$$ (2)

$$A_{i}=\sum_{j\in \left\{d_{ij}\leq d_{0}\right\}}\;G\left(d_{ij}\right)R_{j}$$ (3)

Where *i*, *k* denote the demand points;*R*_*j*_ is the supply-demand ratio;*S*_*j*_ is the sum of supply; *G*(*d*_*ij*_, *d*_0_) is a Gaussian decay function considering the spatial friction problem; *d*_*kj*_ is the cost of service between the supply side *j* and the demand side *k*; *d*_0_ is the set cost of service threshold; *D*_*k*_ is the sum of demand of all demand sides (*d*_*kj*_ ≤ *d*_0_). *d*_*ij*_ is the cost of service between demand sides *i* and *j*; *A*_*i*_ is the accessibility of *i*, the higher the value of *A*_*i*_, the better the accessibility.

#### Improvement of G2SFCA method

To further enhance the accuracy of the model calculation results, this paper improves the traditional G2SFCA method from the aspects of data source, OD cost calculation rules and model parameter optimization:

(1) For the service cost *d*_*ij*_ in the above equation, Euclidean distance was mostly used in previous studies. In this research, the actual distance cost between UPGSs and residential areas is obtained through the network analysis in ArcGIS. In previous studies, supply points were commonly represented by the geometric center of park green spaces. However, due to variations in the size and shape of UPGSs, this approach could lead to significant errors. In this study, for large parks (such as comprehensive and community parks), the real entrances are extracted as the supply points of UPGSs. For small parks (such as gardens and some specific parks) without clear entrances, the geometric center is still used as the supply point, which to some extent improves the accuracy of the data.

(2) With the increasing traffic congestion and parking difficulties in urban central areas, and the popularity of shared bicycles, walking and cycling have gradually become the main choices for travel. Combined with the requirements of UPGS classification in China’s “Urban Residential Area Planning and Design Standards (GB50180-2018)” and “Urban Green Space Planning Standards (GB/T 51346–2019)”, UPGS can be divided into city level, 15-minute living circle level (15-min LC level), 10-minute living circle level (10-min LC level), and 5-minute living circle level (5-min LC level). In addition, it has been found that residents who spend about 20 minutes in a park can significantly improve their mental and physical health, and enhance their sense of well-being and life satisfaction [60, 61]. The concept of the “20-minute park effect” is also popular. In any case, it would be a bad experience for residents to spend more time on the road than in the park. Therefore, the maximum time cost for residents to the park should be limited to 20 minutes. Combined with the concept of green and healthy traveling, we only consider walking within 5-min, 10-min and 15-min service radius, and we consider riding beyond 15-min service radius. We choose walking time cost and bicycle travel time cost to calculate the accessibility of park green space and set the service radius and time cost threshold of different levels of UPGS (Tables 2 and 3) as the basis for subsequent calculation of the distance decay coefficient. The walking time cost and bicycle travel time cost were calculated based on the ratio of the actual distance cost to the corresponding speed. Generally, it is believed that the average speed of riding a standard bicycle on roads is about 15 km/h, and the average walking speed is about 5 km/h.

**Table 2:** Multilevel service radius of the supply side.

Service radius(mins)	UPGS level	Area(ha)
20(ride>1000m)	City level	>10
15(walk≤1000m)	15-min LC level	(5,10]
10(walk≤500m)	10-min LC level	(1,5]
5(walk≤300m)	5-min LC level	(0.2,1]

**Table 3:** Range of the multilevel reach of the demand side.

Range of the reach(mins)	Up to the level of UPGS
(0,5]	City park, 15-min LC park, 10-min LC park, 5-min LC park
(5,10]	City park, 15-min LC park, 10-min LC park
(10,15]	City park, 15-min LC park
(15,20]	City park

(3) In the traditional G2SFCA method, the supplier only considers the quality of the UPGSs itself and does not pay enough attention to the impact of the surrounding environment. Additionally, on the demand side, due to varying subjective preferences among residents, the probability of residents visiting different parks also varies. Therefore, this study mainly makes improvements from both the supply and demand aspects:

1) Supply improvement: In daily use, most residents prefer to visit UPGSs with rich surrounding service functions, that is, the visit rate of residents is positively correlated with the richness of the surrounding functions of UPGSs [62]. The Shannon-Wiener diversity index was originally used to assess the level of biodiversity in the ecological field and has since been widely used in other fields to quantitatively describe the diversity of the composition of study subjects [63]. Points of interest (POIs) are point data with spatial information (such as geographical coordinates, addresses) and attribute information (such as names and categories) [64]. The type, quantity, and density of POIs can effectively reflect the spatial distribution of resident activities. Therefore, this study introduces the Shannon-Wiener diversity index formula and uses urban POI data to calculate the diversity of service functions within the service range of UPGSs (referred to as the diversity of service around UPGSs) *H*_*j*_:

$$H_{j}=-\sum_{j=1}^{m}\;P_{ji}\;\text{ln}\;\left(P_{ji}\right)$$ (4)

Where *H*_*j*_ is the diversity within the service range of UPGS *j*; *m* is the number of POI categories; *i* is a certain category of POIs; *P*_*ji*_ is the proportion of the quantity of *i* category POIs within the service range of UPGS *j* to the total quantity of POIs within the same range of that category. The higher the diversity index value, the more diverse the service functions around the UPGSs are.

Through the supply improvement of the traditional G2SFCA model, the attractiveness *S*_*j*_ of park green space *j* will be determined by two factors: park area and diversity of service around the UPGSs:

$$S_{j}=\left(\gamma_{A}\frac{S_{j}^{\prime }}{\sum_{j}\;S_{j}^{\prime }}\right)+\left(\gamma_{B}\frac{H_{j}}{\sum_{j}\;H_{j}}\right)$$ (5)

Where *γ*_*A*_ and *γ*_*B*_ respectively reflect the importance of UPGS area ($S_{j}^{\prime }$) and the diversity of service around the UPGSs (*H*_*j*_) to the attractiveness (*S*_*j*_), where *γ*_*A*_ = *γ*_*B*_ = 0.5.

2) Demand improvement: According to Park’s research, the richness of UPGSs, the surrounding environment, and the user’s psychological tolerance for distance are the three main factors that affect residents’ choice to visit the UPGSs [65]. Given that residents’ subjective choices can affect the accessibility of UPGSs, this study will calculate the probability of residents’ subjective choice of UPGSs through the Huff model. The Huff model, proposed by David Huff in the United States, is used to calculate the ratio of the attractiveness of a certain service facility to the sum of the attractiveness of all similar facilities in the study area, that is, the probability that residents choose a certain facility [59]. The Huff model is introduced into the G2SFCA model, and the formula is as follows:

$$Q_{ij}=\frac{S_{j}G\left(t_{ij},t_{0}\right)}{\sum_{i\in \left\{t_{ij}\leq t_{0}\right\}}\;S_{j}G\left(t_{ij},t_{0}\right)}$$ (6)

Where *Q*_*ij*_ is the probability that resident *i* chooses UPGS *j* based on Huff’s model; *S*_*j*_ is the attractiveness of *j*; *G*(*t*_*ij*_, *t*_0_) is a Gaussian decay function considering the spatial friction problem; *t*_0_ is the set time cost threshold.

#### Accessibility of UPGSs

(1) Calculation of the supply-demand ratio based on the minimum service standard.

Combining the diversity of service around UPGSs and the Huff model, the improved total supply-demand ratio *R*_*j*_ was calculated. The total supply-demand ratio *R*_*j*_ is the weighted sum of the park’s area supply-demand ratio $R_{j}^{\prime }$ and the service diversity supply-demand ratio $H_{j}^{\prime }$. *H*_*j*_ is dimensionless, so its value can replace $H_{j}^{\prime }$. Since the minimum service standards for $R_{j}^{\prime }$ and $H_{j}^{\prime }$ are different, they cannot be summed directly and must first be normalized. The value of $R_{j}^{\prime }$ was normalized by dividing it by the minimum per capita public green space area (*R*_0_) of 12m^2^ in the built-up area specified in the Chinese garden city standard, and the value of $H_{j}^{\prime }$ was normalized by dividing it by the minimum park green space diversity index *H*_0_ in the study area. Since there is no standard value for the minimum park green space diversity index *H*_0_, it is measured by the median value of *H*_*j*_, which is 2.15.

$$R_{j}^{\prime }=\frac{M_{j}}{\sum_{i\in \left\{t_{ij}\leq t_{0}\right\}}\;Q_{ij}D_{i}}$$ (7)

$$R_{j}=\left(\gamma_{A}\frac{R_{j}^{\prime }}{R_{0}}\right)+\left(\gamma_{B}\frac{H_{j}^{\prime }}{H_{0}}\right)$$ (8)

Where *R*_*j*_ is the supply-demand ratio of UPGS *j*; $R_{j}^{\prime }$ is the supply-demand ratio of UPGS *j* without considering the diversity of surrounding services; *M*_*j*_ is the area of UPGS *j*; *Q*_*ij*_ is the probability of resident *i* choosing UPGS *j*; *D*_*i*_ is the population of residential area *i* within the scope of UPGSs (*t*_*ij*_ ≤ *t*_0_). *R*_0_ is the minimum per capita public area of 12m^2^; *H*_0_ is the minimum UPGS diversity index of 2.15.

(2) Accessibility calculation. The supply-demand ratio is weighted using the probability of residents’ subjective choices, and then the weighted supply-demand ratios

*R*_*j*_ are summed to obtain the accessibility *A*_*i*_ for each residential area:

$$A_{i}=\sum_{j\in \left\{{\text{t}}_{ij}\leq t_{0}\right\}}\;Q_{ij}R_{j}$$ (9)

The improved G2SFCA model of this study comprehensively considers factors such as the real supply points of UPGSs, residents’ subjective choice probabilities for parks, travel time impedance for residents to different types of parks, and the diversity of service around UPGSs.

#### Equity evaluation method

(1) Spatial equity evaluation method

Based on the accessibility evaluation results, the spatial equity of UPGS supply-demand is measured:

$$E_{i}=A_{i}\times \frac{\text{max}\left(R_{j}\right)}{\text{max}\left(A_{i}\right)}$$ (10)

Where *E*_*i*_ is the spatial equity value, which represents the supply-demand matching relationship between UPGSs and residents. According to the research of Taleai, M et al., the calculation results are divided into six levels (Table 4) [55], which are high, sufficient, balance, undersupply, shortage, and no supply.

**Table 4:** Classification of supply-demand levels.

Accessibility Level	Value Range	Supply-Demand Situation
Very high	*E*_*i*_ > 1.00	High
High	0.75 ≤ *E*_*i*_ ≤ 1 .00	Sufficient
Medium	0.50 ≤ *E*_*i*_ ≤ 0.75	Balance
Low	0.25 ≤ *E*_*i*_ < 0.50	Undersupply
Very low	0 < *E*_*i*_ < 0.25	Shortage
No	*E*_*i*_ = 0	No supply

In order to intuitively reflect the spatial distribution patterns of UPGS accessibility(*A*_*i*_) and supply-demand equity(*E*_*i*_), we used the untransformed empirical Bayesian Kriging method to interpolate the discrete point data into continuous surface data.

(2) Social equity evaluation method

Lorenz curve and Gini coefficient are statistical methods used to evaluate the inequality of income or wealth distribution, which are mainly used in economics and sociology. Since the Lorenz curve and Gini coefficient can reflect differences in resource distribution among different groups and then analyze social inequality, they are now widely used in evaluating the equity of urban facility distribution [39, 66, 67]. This study also uses the Lorenz curve and Gini coefficient to evaluate the social equity of urban park supply-demand. The Lorenz curve depicts income or wealth inequality through the relationship curve between population (%) and income or wealth (%). In this study, the Lorenz curve, represented by the permanent population and UPGS accessibility, reflects the inequality in urban park resource distribution. A Gini coefficient of 0.4 is often considered the warning line of distribution inequality. According to the evaluation standards of the United Nations Development Programme, a Gini coefficient between 0.3 and 0.39 is considered “relatively reasonable,” while a coefficient between 0.4 and 0.59 indicates a “large disparity.” We rank the districts in the study area according to the accessibility of urban park resources per capita from low to high, and calculate the proportion of permanent residents in urban park resources in each 10% interval. The Gini coefficient is calculated from the Lorenz curve. The formula is as follows:

$$G=1-\sum_{k=1}^{n}\left(P_{k}-P_{k-1}\right)\left(A_{k}-A_{k-1}\right)$$ (11)

Where *G* is the Gini coefficient; *P*_*k*_ is the cumulative percentage of the permanent population, and *A*_*k*_ is the cumulative percentage of UPGS accessibility. The value of the Gini coefficient G ranges between 0 and 1, where G = 0 means complete equity, and G = 1 means complete inequity. The greater the Gini coefficient, the more inequality the distribution of UPGSs; the smaller the Gini coefficient, the more equitable the distribution of UPGSs.

#### UPGS layout optimization method

There are seven different types of LA models to determine the logical location of facility points in ArcGIS, four of which are related to public service facilities: the minimum impedance model, the maximum coverage model, the maximum capacitated coverage model, and the minimum facilities model. Their main features and application scenarios are shown in Table 5.

**Table 5:** The LA models for public service facilities.

LA Models	Main Features	Number of Facilities	Application Scenarios
Minimum impedance	Minimize the sum of weighted costs between the request points and the facility points	Known	Usually applied to non-emergency public facilities that prioritize efficiency
Maximum coverage	As many request points as possible are allocated within the impedance interruptions of the solved facility points	Known	Usually applied to emergency public facilities that meet specified response times
Maximum capacitated coverage	Similar to the maximum coverage model, but the weighted requests allocated to facility points cannot exceed the capacity of the facility points	Known	Usually applied to public facilities with limitations on the number of personnel or business transactions
Minimum facilities	Similar to the maximum coverage model, but with the requirement to minimize the number of facility points covering the request points	Unknown	Considered the construction cost of facility points based on the maximum coverage model

Source:Draw based on the usage instructions for the LA model in the ArcGIS help documentation.

Due to the varying service utilities of UPGSs of different scales in the city, the appropriate LA model should be chosen for layout optimization based on their objectives. City level and 15-min LC level UPGSs have a large service range, but the construction cost is high. In this case, priority should be given to maximizing service coverage while minimizing overlap, and the minimum facilities model should be chosen. The 10-min LC level and 5-min LC level UPGSs mainly emphasize the convenience for urban residents, where the weighted impedance sum between residential areas and UPGSs should be minimized. Therefore, we should choose the minimum impedance model and combined with the minimum facilities model for multi-scheme selection.

## Results

### Accessibility analysis of UPGS distribution

**Fig 4:**
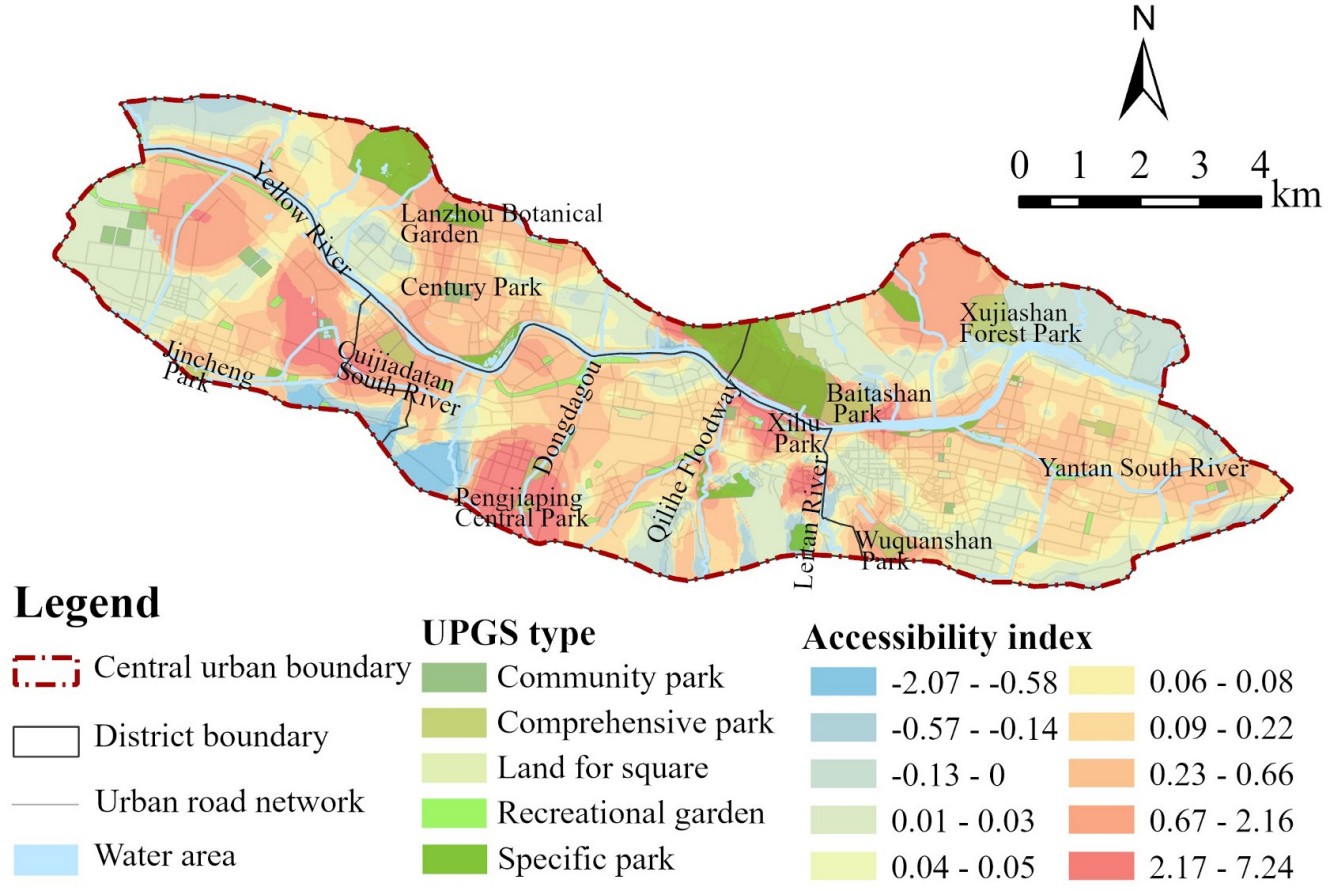
Trend analysis of the accessibility index in improved G2SFCA model.

As shown in Fig 4, on the whole, the accessibility of UPGSs in the central urban area of Lanzhou has obvious distribution characteristics along the river. The areas with high accessibility index are mainly located along the Yellow River, the middle of Anning District, Cuijiadatan South River, Dongdagou, Qilihe Floodway, Leitan River, Yantan South River and other areas. However, there are significant differences in the accessibility of UPGS in local areas. For example, Lanzhou Botanical Garden, Century Park, Pengjiaping Central Park, Xihu Park, Baitashan Park, Wuquanshan Park, etc., have better accessibility due to their location in urban areas, abundance of surrounding service facilities, and convenient transportation, which increases residents’ willingness to choose them. Conversely, Xujiashan Forest Park, Jincheng Park, and others, either due to inconvenient surrounding transportation or being located on the edge of urban areas with fewer surrounding service functions, have lower resident visitation willingness, resulting in poorer accessibility.

In summary, the current UPGSs in the central area of Lanzhou City are mainly distributed along the river system. The accessibility of UPGSs has obvious “string of beads” distribution characteristics. The areas with high accessibility index are mainly distributed along the river and in some peripheral areas of the central city, especially along the Yellow River. In other areas, UPGSs are less distributed, resulting in an overall imbalanced distribution and poor equity. The Areas with low matching between supply and demand urgently need to optimize their layout to ensure that all citizens have equal rights to public service facilities.

### Equity evaluation of UPGS supply-demand

#### Social equity

**Fig 5:**
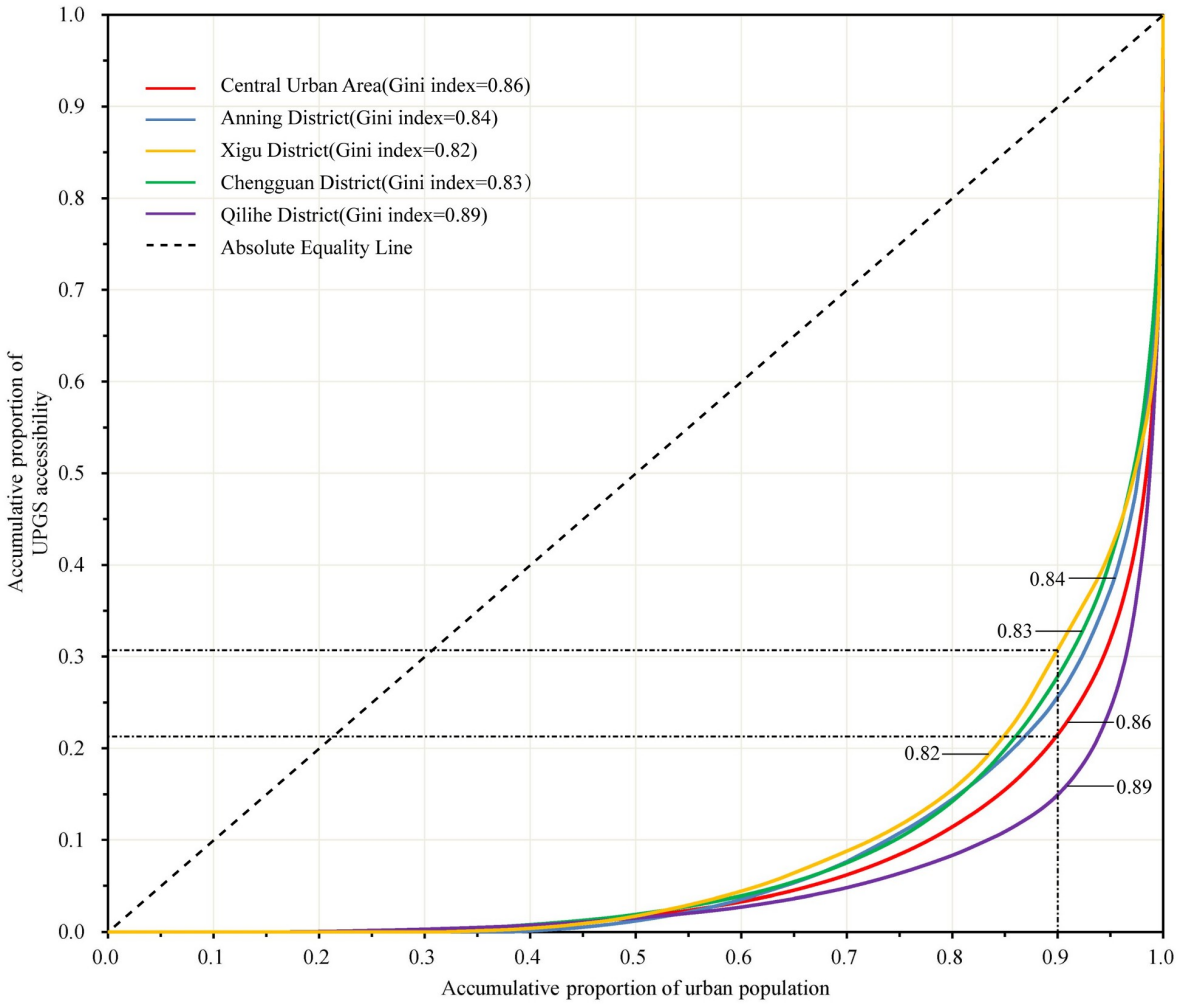
Lorenz curve for social equity of UPGS distribution.

In the central urban area of Lanzhou City, the Gini coefficient for the distribution of UPGS resources among all residents is 0.86, far exceeding the warning line of 0.4, indicating a significant disparity in resource distribution. The Gini coefficient of each district from large to small is: Qilihe District (0.89) > Anning District (0.84) > Chengguan District (0.83) > Xigu District (0.82) (Fig 5). Similarly, the Gini coefficient of each district is also much larger than the warning line of 0.4, showing a significant inequity in the distribution of UPGS resources. Further analysis of the Lorenz curve reveals that 90% of the residents in the central urban area of Lanzhou can only access 22% of the total UPGS resources. Even in Xigu District, which has a relatively lower Gini coefficient, 90% of its residents can only use about 30% of the total UPGS resources in the district. This shows that more UPGS resources are enjoyed by a few people.

#### Spatial equity

**Fig 6:**
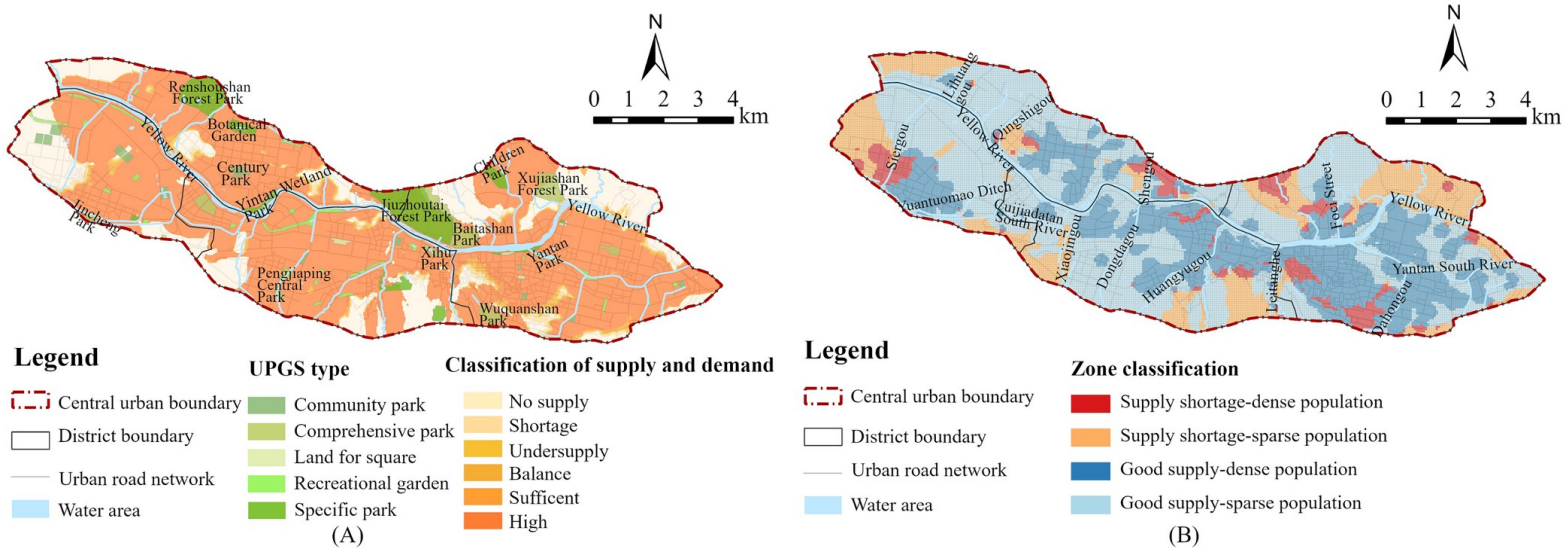
(A) Supply-demand classification; (B) Supply-demand and population classification.

To further analyze the supply-demand relationship between UPGSs and residents, the supply-demand evaluation was conducted based on the accessibility results. It was found that the UPGS supply-demand in the central urban area of Lanzhou City differed significantly in the area proportion and spatial distribution (Table 6, Fig 6A). The areas with “high”, “sufficient” and “balance” in the whole study area were mainly located along the Yellow River and its tributaries, accounting for 74.33%, 1.10%, and 1.27% of the area, respectively. Overall, the areas with balance and above accounted for 76.70% of the total central urban area of Lanzhou, and there were still 23.30% of areas with unsatisfactory supply-demand conditions. Combined with population density, the study area can be classified into four categories: good supply—dense population, good supply—sparse population, supply shortage—dense population, and supply shortage -sparse population (Fig 6B). The supply shortage-dense population areas are prioritized for future planning. The supply-demand were evaluated by *E*_*i*_ value where *E*_*i*_ ≥ 0.5 indicates well-supplied, and *E*_*i*_ < 0.5 indicates a supply shortage. The population was defined by the average population density value (about 12,700 people/km^2^), with values above average considered dense population and below average considered sparse population. The analysis showed that the overall supply situation exhibits a circle distribution pattern, from the outside to the inside: supply shortage—sparse population, supply shortage—dense population, good supply—sparse population, good supply—dense population. The supply-demand situation of each area was analyzed in detail by combining the UPGS configuration and the diversity of surrounding services.

**Table 6:** Area proportion of different supply-demand levels in different areas.

Sort	Total/%	Anning District/%	Chengguan District/%	Qilihe District/%	Xigu District/%
High	74.33	78.68	69.60	80.52	70.31
Sufficient	1.10	1.27	1.39	0.72	0.85
Balance	1.27	1.53	1.73	0.72	0.82
Undersupply	1.32	1.51	1.85	0.79	0.74
Shortage	1.97	2.14	2.93	1.17	0.92
No supply	20.01	14.87	22.50	16.07	26.36
Total	100.00	100.00	100.00	100.00	100.00

1. Xigu District, poor supply-demand. The proportions of “no supply”, “shortage” and “undersupply” areas in Xigu District were 26.32%, 0.92%, and 0.74% respectively. The total proportion of the three areas (supply shortage areas) was 28.02%, which was the largest proportion among the four jurisdictions in the central urban area of Lanzhou. Conversely, the total area of the three areas of “balance”, “sufficient” and “high” (good supply areas) accounted for 71.98% of the area of Xigu District, which was the smallest among the four jurisdictions in Lanzhou’s central urban area. Additionally, the proportion of “no supply” area was the largest among the four jurisdictions in the central city of Lanzhou. The areas with good supply were mainly located the south of the Yellow River, the north of Si’ergou and the northwest of Yuantuomao Ditch. These areas were distributed in Jincheng Park, Xigu Urban Sports Park and a certain number of community parks and recreational gardens. The layout of various parks was relatively scattered, and the number was relatively large. The supply shortage areas were mainly located in the southwest and southeast. As Xigu District undertook the main industrial functions of Lanzhou City, many industrial enterprises (such as Lanzhou Petrochemical Company) were mainly located in the north, and the south is mainly its living area. Therefore, the population density in the southern region was relatively high, especially in the southwest, where the supply-demand contradiction was more prominent and supply was scarce. The areas with supply shortage-dense population density were mainly distributed on both sides of the southern section of Si’ergou.
2. Chengguan District, relatively poor supply-demand. The proportions of “no supply”, “shortage” and “undersupply” areas in Chengguan District were 22.50%, 2.93% and 1.85% respectively. The total proportion of the three areas (supply shortage areas) was 27.28%, only second to Xigu District among the central urban districts, showing a challenging supply-demand situation. The good supply areas accounted for 72.72% of the area of Chengguan District. The good supply areas were mainly distributed along the Yellow River, Yantan South River and the northern area of Foci Street. The supply shortage areas were mainly located on the periphery of Chengguan District, showing “⊃” type distribution pattern. The supply shortage-dense population situation was mainly distributed on the west side of Foci Street and Dahonggou. There were large parks such as Jiuzhoutai Forest Park, Baitashan Park, Children Park, Xujiashan Forest Park, Yantan Park and Wuquanshan Park in Chengguan District, which was the largest number of the four jurisdictions in the central urban area of Lanzhou. However, the supply shortage-dense population areas of Chengguan District were also the largest in the four jurisdictions of Lanzhou, and the distribution was scattered. In addition, the number of recreational gardens was insufficient, which led to the shortage of UPGSs, especially the fewer small community parks and recreational gardens in the northern part of Chengguan District.
3. Anning District, relatively good supply-demand. The proportions of “high”, “sufficient” and “balance” areas in Anning District were 78.68%, 1.10% and 1.27% respectively. The total proportion of the three areas (good supply areas) was 81.48%, which is close to the situation of Qilihe District. However, the supply shortage areas in Anning District accounted for 18.52% of its total area. The good supply areas were primarily located in the central part of Anning District. This area included two large specific parks, Renshoushan Forest Park and Botanical Garden, and a larger community park, Century Park. Moreover, the surrounding facilities of these UPGSs were relatively complete and the service functions were diverse, resulting in a relatively good supply-demand situation in this area. In addition to the above three large park green spaces, the number and area distribution of small community parks, recreational gardens and other specific parks in the Anning District is less or even almost non-existent, which also led to a shortage of supply in the eastern and western areas of Anning District. The areas with supply shortage-dense population were mainly located on both sides of Qingshigou and Shengou.
4. Qilihe District, good supply-demand. The proportions of “high”, “sufficient” and “balance” areas in Anning District were 80.52%, 0.72% and 0.72% respectively. The total proportion of the three areas (good supply areas) was 81.96%, which was the highest proportion of the four jurisdictions in the central urban area of Lanzhou. Conversely, the proportion of supply shortage area in Qilihe District was 18.04%, which was the lowest among the four jurisdictions in Lanzhou Central City. The areas with good supply were mainly located south of the Yellow River, both sides of the south channel of Cuijiadatan, both sides of Dongdagou, the northwest of Huangyugou and the northwest of Leitan River. In these areas, UPGS types were rich, the number was relatively large and the layout was relatively scattered. The services surrounding these UPGSs were convenient and diverse, facilitating resident access. The supply shortage areas were mainly located in the southwest and southeast, and the supply shortage-dense population areas were also relatively scattered. In the future planning of these areas, priority should be given to community parks and recreational gardens.

In summary, the supply-demand contradiction of UPGSs in the central urban area of Lanzhou is still prominent. The main reasons are the small number of UPGSs, insufficient area, single type of local areas, and unreasonable layout. To effectively meet the needs of residents and improve their quality of life, it is urgent to supplement and optimize the UPGSs in the central urban area of Lanzhou.

### UPGS layout optimization

A good location for UPGSs not only ensures low operational and maintenance costs of parks, but also maintains high accessibility and utilization rates, providing high-quality services to the city at a lower cost. The LA model in ArcGIS can provide solutions for the siting and layout optimization of UPGSs. The LA model contains three main parameters: demand points, routes, and facilities. In this study, the demand points are all residential areas, the candidate facilities are new UPGSs, and the routes are network paths between UPGSs and residential areas under different impedance interruptions. In addition to prioritizing highly accessible plots and maintaining the traditional planning pattern of Lanzhou, the candidate location selection for these new UPGS must also consider the following important factors:

Firstly, supply shortage areas, especially supply shortage-dense population areas, should be given priority.

Secondly, Lanzhou is a river valley city where the Yellow River runs through the city, and the north and south are surrounded by mountains. To further improve the accessibility of UPGSs and highlight the city’s mountain-river characteristics, the candidate location of new UPGSs should be fully integrated with the river system. In particular, it emphasizes the construction of waterfront green belts along the Yellow River and its branch water systems to create an urban “blue-green” park system with balanced supply-demand.

Thirdly, the candidate location for new UPGS should be based on the intensive land use and easy access to the public. The candidate location of the new UPGS are mainly the planned green spaces and unused lands in the “Lanzhou City Master Plan (2011–2020)”, as well as inefficient stock lands determined through a combination of Google Earth imagery and field surveys, as outlined. At the same time, we have also considered the optimized layout of future urban roads in the “Lanzhou ‘14th Five-Year’ Urban Transportation Development Plan”.

**Fig 7:**
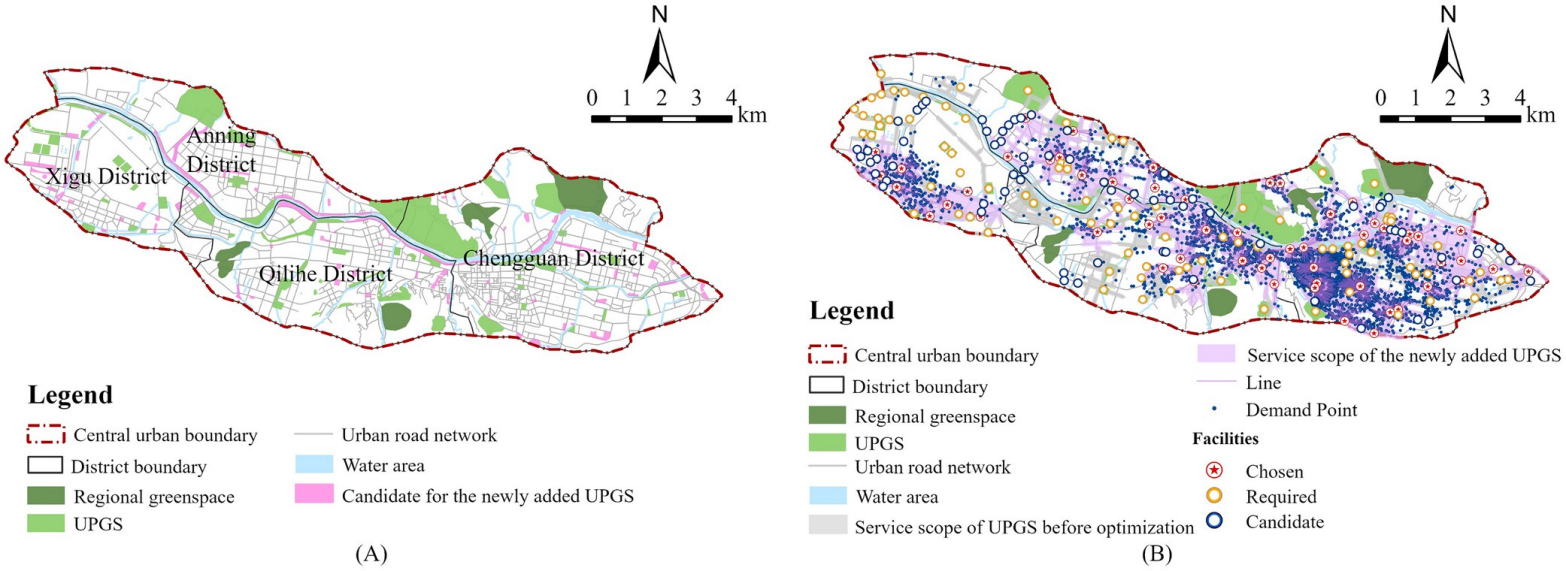
(A) The distribution of the chosen candidates; (B) The distribution of the newly added UPGSs.

Considering the above factors, we finally identified 126 possible candidate locations for new UPGS in the future. Due to China’s current advocacy of “progressive micro-regeneration” in urban renewal actions strictly controls large-scale additions and demolitions, the 126 new UPGS candidate facilities in this article are community parks and UPGSs at the following levels (Fig 7A).

**Fig 8:**
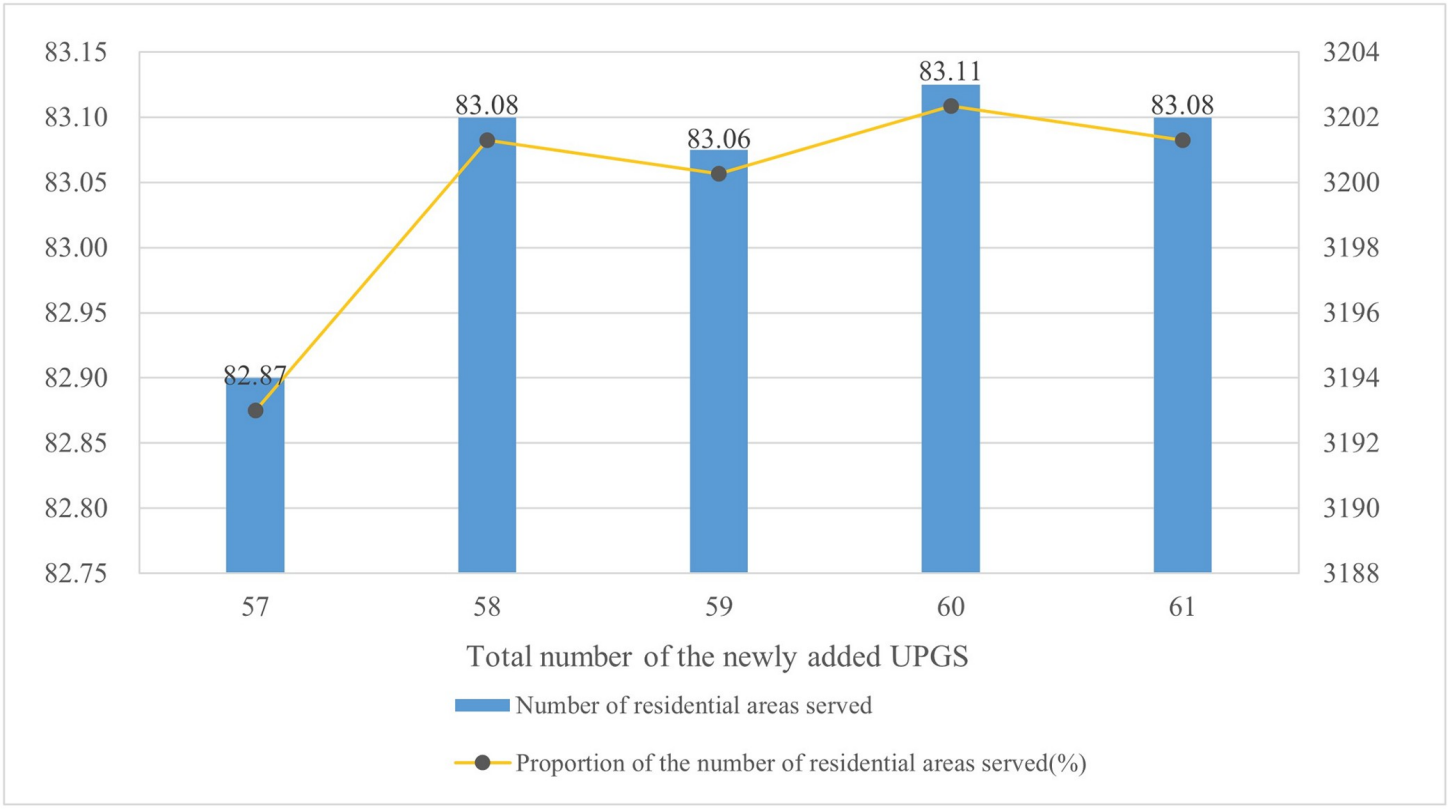
Simulation results of multiple schemes based on the minimum impedance model.

The International Ecocity Standards (www.ecocitystandards.org) emphasize the concept of “close proximity access” to facilitate citizens to access green spaces through walking. Similarly, the planning concept of “walking-minute living circle” has been actively promoted in China. To create a safe, sanitary, convenient, comfortable, and livable residential environments, China’s “Urban Residential Area Planning and Design Standards (GB50180-2018)” (http://www.moe.gov.cn/jyb_xwfb/xw_zt/moe_357/jyzt_2019n/2019_zt13/zcwj/201906/t20190606_384732.html) divides the residential areas into four levels: the 15-minute living circle residential area, 10-minute living circle residential area, 5-minute living circle residential area, and residential neighborhood. The highest level of residential area is the 15-minute living circle residential area, where all essential services are accessible within a 15-minute walk. In ArcGIS, we established a location-allocation model for new UPGSs. The demand points consisted of the existing 3,853 residential areas, the facility points were the candidate location for the 126 new UPGSs, and the routes were derived from the previously established urban road network dataset, with a maximum impedance cutoff value of 15 minutes. First, using the minimum facilities model, we calculated that the minimum number of new UPGS needed to cover most residential areas was 58. Moreover, based on the above standards, the minimum impedance model was used to calculate the number of residential areas covered by 57, 58, 59, 60, and 61 candidate UPGSs respectively. Finally, by comparing the number of served residential areas and the proportion of served residential areas for multiple scenarios, the service benefits of the candidate UPGSs was characterized (Fig 8). Finally, it was determined that the selected 58 UPGSs represented the best solution (Fig 7B).

## Discussion

### Influence of different factors on equitable evaluation of UPGS supply-demand

#### Influence of park quality and residents’ willingness on UPGS accessibility

An important indicator to measure whether the spatial configuration of UPGSs is reasonable is the accessibility of residents. Previous studies mostly used the G2SFCA method and its improvements to measure the accessibility of UPGSs, but ignored the residents’ willingness to visit park and the quality of park [47, 68, 69]. Usually, park quality and travel costs are the main factors affecting residents’ willingness to choose. The quality of the park is mainly related to the size of the park, the diversity of the internal facilities and the surrounding facilities. The travel costs mainly include distance costs and time costs. In fact, residents in different countries and regions will weigh the travel costs of different transportation modes based on their travel habits, travel purposes, management policies and other factors to choose suitable parks. Combining China’s planning concept of “minute living circle residential areas” and the increasing popularity of shared bicycles in cities, this study has improved the G2SFCA method. It not only considers the time cost thresholds for walking and cycling in different levels of living circles, but also considers the probability of residents’ subjective choice to visit the parks and the diversity of service around the park. The research results confirmed that parks such as Baitashan Park, Xihu Park, Pengjiaping Central Park, and Lanzhou Botanical Garden, which have larger areas and a variety of surrounding facilities, also have higher accessibility. Most previous studies took subdivisions or communities as research units [70, 71]. Larger administrative boundaries can increase errors in population data and travel time estimates. In this study, the residential area is used as the study unit, which has a finer study scale, more accurate calculation and simulation results. It is more helpful to identify the spatial differentiation characteristics of the UPGS layout.

#### Influence of population density and linear urban form on UPGS supply-demand equity

The essence of UPGS supply-demand equity research is the matching relationship between UPGS spatial distribution (supply side) and residential population (demand side).

Fuller, R.A. et al. found that there are significant differences in the distribution of UPGSs and per capita green space in different areas of the city, and the supply of UPGSs has little to do with the city’s area but more to its natural geographical pattern and population [72]. This indicates that urban form and population density have a great impact on the UPGS supply-demand. Currently, most studies focus on grid or grid + radial urban layouts, with less attention to linear large cities. Taking Lanzhou, a typical representative of high-density linear cities, as an example, this paper analyzes the impact of population density and linear urban form on UPGS supply-demand equity through quantitative methods. The results show that in terms of social equity, whether from the city level or the district level, the resource allocation gap of UPGS is very large, and 90% of the residents can only enjoy about 20% of the UPGS resources. In terms of spatial equity, the supply-demand status in different areas also have significant differences. At the city level, about 20% of areas are still in shortage of UPGS supply, and the areas with supply shortage are mainly located on the periphery of the city, far away from the Yellow River. The UPGSs with high accessibility is mainly concentrated along the Yellow River and its tributaries, showing obvious “string of beads” distribution Characteristics. This distribution is due to the roads in valley linear cities often running parallel to rivers, with parks also arranged along these waterways.

**Fig 9:**
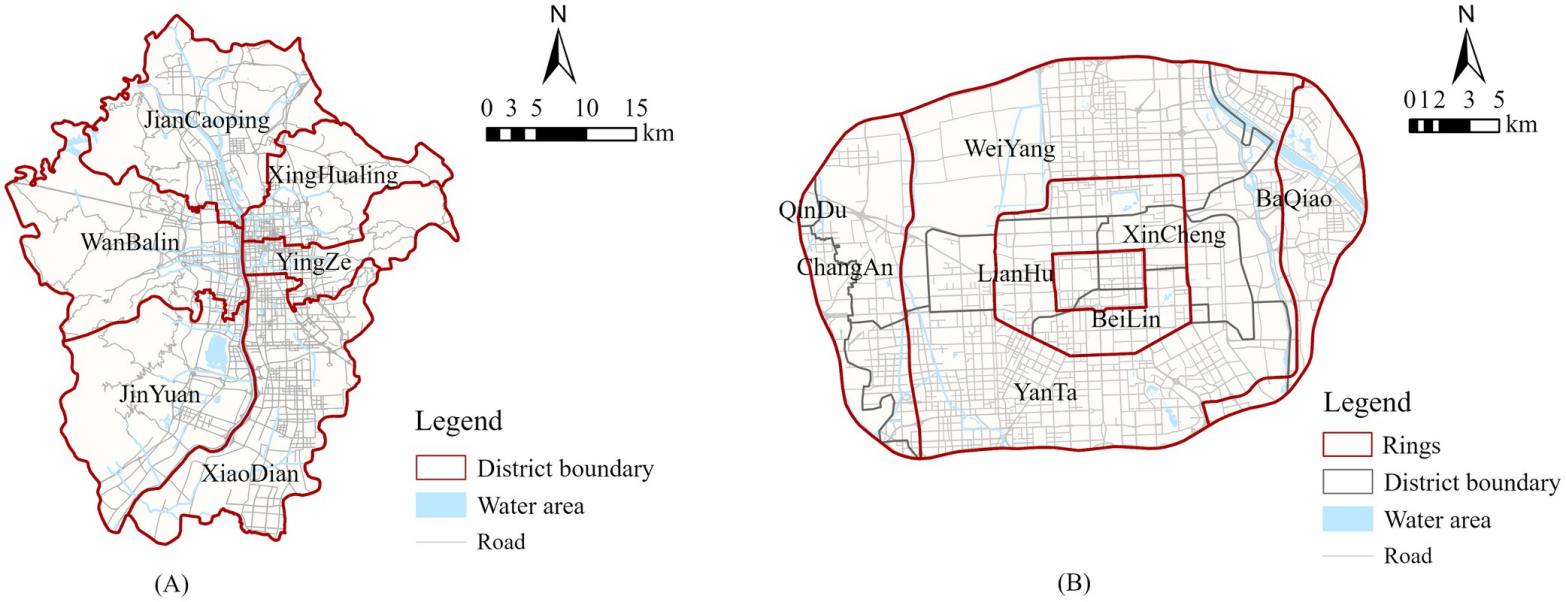
(A) Taiyuan morphological map; (B) Xi’an morphological map.

Combined with population density, the study found that the supply-demand of UPGSs in Lanzhou showed a spatial gradient effect. From the city center to the periphery, the order was: good supply—dense population, good supply—sparse population, supply shortage—dense population, supply shortage—sparse population. At the district level, the supply-demand of UPGSs in Lanzhou City from strong to weak was: Qilihe District > Anning District > Chengguan District > Xigu District. Lanzhou City is surrounded by mountains in the north and south, and the Yellow River passes through the middle of the city from west to east. The North and South Riverside Roads along the Yellow River are the city’s main east-west traffic arterial road, while the north-south arterial roads are laid out perpendicular to the Yellow River. The traffic flow in the city eventually converges on the North and South Riverside Road. The unique geographical pattern has created Lanzhou’s distinctive “fishbone” trunk road network structure and a linear urban form with the Yellow River as the development axis. The study found that the “fishbone” trunk road network structure is an important factor leading to the spatial gradient effect of UPGS supply-demand. The uneven distribution of UPGSs and population is another distinctive feature of linear cities. The UPGSs are mostly concentrated along the Yellow River in the city center, resulting in the good supply of UPGSs in the city center and the poor supply of UPGSs in the peripheral areas. In order to reflect the UPGS supply-demand differences between the linear large cities and those with grid and radial-circular layouts, we took Taiyuan (grid layout) and Xi’an (grid + circular radiation layout) as examples to illustrate (Fig 9), which are cities of the same size class in northern China. According to the existing studies [73, 74], more than 90% of residents in the above two cities can enjoy more than 40% of UPGS resources, which is much higher than the linear city Lanzhou. Similar to Lanzhou, there is a trend of gradual decrease from the center of the city to the periphery in the spatial accessibility of UPGSs. However, the spatial accessibility of the linear city Lanzhou’s UPGS shows an unbalanced rectangular trend, decreasing from the central axis towards the outer areas. In contrast, the UPGS spatial accessibility of grid and grid + circular radial cities show a relatively balanced square or circular trend, which gradually decreases from the center to the periphery.

### UPGS layout optimization under the condition of limited urban land resources

The land resources in the center of high-density cities are generally very limited, and it is not easy to ensure that all the people in the city can enjoy good environmental resources equitably by adding new UPGSs. In order to make the UPGS layout optimization more scientific, we have established the working concept of “preliminary selection of candidate location for new UPGSs—schemes simulation and comparison—optimal scheme determination”. In the stage of “preliminary selection of candidate location for new UPGSs,” we formulated the priority principles of “supply shortage—dense population area,” “highlight city’s mountain-river characteristics” and “intensive use of land and easy access for citizens,” and also integrated the concept of “progressive micro-regeneration.” On this basis, we simulated and compared the new UPGS candidate locations through the LA model in ArcGIS and determined the final new UPGS location based on the principle of maximizing service benefits. The above work ideas and principles provide a scientific method for UPGS layout optimization.

### Limitations

There are still some limitations in this study. First of all, when assessing the service capacity of UPGSs, we only considered the parks’ area and the diversity of surrounding services, neglecting the quality of the internal landscape environment and service facilities. Secondly, different social groups also have differences in the equitable enjoyment of the right to use UPGSs [1, 15]. This article only focuses on the general population. However, specific groups such as children, the elderly and the disabled have special needs for UPGSs. To their equitable access to UPGSs, the impact of accessibility on UPGS accessibility needs to be considered in the future. This article only focuses on the general population. However, specific groups such as children, the elderly and the disabled have special needs for UPGS. To ensure their equitable access to UPGS, the impact of barrier-free environments on the accessibility of UPGSs needs to be considered in the future. Thirdly, the most significant challenge for valley-type linear cities is traffic problems, which is manifested as traffic congestion along the river. The vertical direction is limited by river separation and valleys, and the traffic links between the two sides of the river are inconvenient. This leads to limited space for urban development in depth. In addition, the vertical direction is affected by river separation and valley constraints, resulting in inconvenient traffic links on both sides of the river and limited urban development space in depth. In the future, the optimization of UPGSs can start from the road system and non-construction land in the periphery of the city. Fourthly, there are morphological differences between linear cities and other cities. The unified use of mainstream methods in equity measurement and optimization layout methods may lead to certain errors, and the optimization costs of different forms of cities may also be different, which need to be further studied in the future. Lastly, this study only selected the typical representative Lanzhou to analyze the supply-demand characteristics of UPGSs in high-density linear cities. To enhance the generalizability of the results, other linear big cities should be selected for verification in the future.

## Conclusions

This study established a research framework of “model improvement—supply- demand equity evaluation—preliminary selection of candidate location for new UPGSs—schemes simulation and comparison—optimal scheme determination.” The improved G2SFCA model considered the subjective choice of residents and the diversity of services around the park. Based on this framework, we assessed the equity of UPGS supply-demand and determined the optimal solution to ensure the maximization of UPGS supply-demand equity. Compared with previous studies, we selected Lanzhou City as a case to reveal the social and spatial inequity characteristics of UPGS supply-demand at both urban and district scales in high-density valley-type linear cities. The research shows that the areas with high spatial accessibility of UPGSs in valley-type linear cities are mainly concentrated along the river, and the supply-demand equity has a spatial gradient effect. From the inside to the outside, the order is: good supply—dense population, good supply—sparse population, shortage supply—dense population, shortage supply—sparse population. The spatial accessibility of UPGSs in linear cities, similar to grid and grid + circular radial cities, has a trend of gradually decreasing from the center to the periphery. However, under the same proportion of population, linear cities can only enjoy a smaller proportion of UPGS resources. This indicates that the social inequality of linear cities is more serious than that of grid cities and grid + circular radial cities. This may be caused by the relatively unbalanced spatial structure of the linear big cities. However, it is also necessary to further analyze the influence of the city’s geographical-climatic conditions, ecological status, economic development and other factors. Lanzhou is located in the arid area of northwest China, with a geographical pattern of two mountains sandwiching a river. It has low vegetation coverage, fragile ecology, and high population density and building density. The land in the central city of Lanzhou is extremely tight. In the future, we should adopt a “progressive micro-renewal” approach and balance efficiency and equity to gradually add green spaces. At the same time, we should also take into consideration the city’s mountain-water characteristics and create a blue-green intertwined park and green space system to ensure that all residents can enjoy the green and healthy park resources equitably.
